# Tumoren der Mundhöhle

**DOI:** 10.1007/s00117-020-00756-5

**Published:** 2020-10-06

**Authors:** R. Rotzinger, B. Bachtiary, A. Pica, D. C. Weber, F. Ahlhelm

**Affiliations:** 1 Abteilung Neuroradiologie, Zentrum für Bildgebung, Kantonsspital Baden AG, Im Ergel 1, 5404 Baden, Schweiz; 2 Zentrum for Protonentherapie, Paul Scherrer Institut, ETH Domain, CH-5232 Villigen, Schweiz

**Keywords:** Otorhinolaryngologie, Malignom, Neoplasma, Plattenepithelkarzinom, Bildgebung, Otorhinolaryngology, Malignancy, Neoplasms, Squamous cell carcinoma, Diagnostic imaging

## Abstract

**Klinisches/methodisches Problem:**

Mundhöhlenmalignome stellen weltweit die häufigsten Tumoren im Bereich der Hals-Nasen-Ohrenheilkunde bzw. Otorhinolaryngologie dar. Es handelt sich um eine heterogene Gruppe an Tumoren, deren Kenntnis erforderlich ist, um den unterschiedlichen Anforderungen an Diagnostik und Therapie gerecht zu werden.

**Radiologische Standardverfahren:**

Computertomographie (CT), Magnetresonanztomographie (MRT), Sonographie, nuklearmedizinische Verfahren (NUK).

**Leistungsfähigkeit:**

Die o. g. Diagnostika werden komplementär eingesetzt.

**Bewertung:**

Eine frühzeitigere Diagnose des Tumors verbessert das Staging und somit die Therapie und Prognose des Patienten.

**Schlussfolgerung:**

Dem Radiologen kommt bei der interdisziplinären Behandlung von Malignomen der Mundhöhle eine bedeutende Rolle zu. Trotz großer Fortschritte in der Radiotherapie, Onkologie und Immuntherapie spielt die Chirurgie weiterhin eine wichtige Rolle in der Behandlung maligner Erkrankungen der Mundhöhle.

## Abstract (en)

**Clinical/methodical issue:**

Oral cavity malignancies are the most common tumors in the field of ear, nose and throat medicine or otorhinolaryngology worldwide. It comprises a heterogeneous group of tumors, the knowledge of which is necessary to meet the different requirements of diagnostics and therapy.

**Standard radiological methods:**

Computed tomography (CT), magnetic resonance imaging (MRI), sonography (US), nuclear medical procedures (NUK).

**Performance:**

The above-mentioned diagnostics are used in a complementary manner.

**Achievements:**

Early diagnosis of the tumor improves staging and thus the patient’s therapy and prognosis.

**Practical recommendations:**

The radiologist plays an important role in the interdisciplinary treatment of malignant tumors of the oral cavity. Despite great progress in radiotherapy, oncology and immunotherapy, surgery still plays an important role in the treatment of malignant diseases of the oral cavity.

## Hintergrund

Gegenwärtig stellen Mundhöhlenmalignome weltweit die häufigsten Tumoren im Bereich der Hals-Nasen-Ohrenheilkunde bzw. Otorhinolaryngologie dar. Es werden über 350.000 neue Fälle pro Jahr diagnostiziert (höchste Inzidenzrate ist in Südasien und den Pazifikinseln). In Indien und Sri Lanka machen Tumoren der Mundhöhle die häufigste Krebstodesrate aus. Gemäß der Global Cancer Statistics 2018 beträgt die Inzidenz in Westeuropa bei Männern 6,9 und bei Frauen 3,2 pro 100.000 Einwohner [1].

Der wichtigste Risikofaktor für die Entstehung ist jede Form von Tabak- und Alkoholkonsum (Ausnahme: Mukoepidermoidkarzinom). Wirken beide Faktoren zusammen, verstärkt sich der Effekt erheblich. Weitere Hauptrisikofaktoren sind chronische Infektionen mit humanen Papillomviren (HPV), der Verzehr großer Mengen von Lebensmittel, die Nitrosamine enthalten (z. B. gepökelter Fisch) oder bei Karzinomen der Lippe direkte UV-Strahlung.

## Anatomie

Die Mundhöhle (*Cavum oris*) ist der Raum, der nach vorn von den Lippen (*Labia*), nach oben durch harten und weichen Gaumen (*Palatum durum et molle*), nach seitlich durch die Wangen (*Buccae*) und nach unten durch den Mundboden (*Diaphragma oris*) begrenzt wird. Nach hinten setzt sich die Mundhöhle kontinuierlich in den Rachen (*Pharynx*) fort und wird begrenzt durch die Rachenenge (*Isthmus faucium*), gebildet durch den vorderen und hinteren Gaumenbogen (*Arcus palatoglossus et palatopharyngeus*).

Zwischen vorderem und hinterem Gaumenbogen liegt die Gaumenmandel (*Tonsilla palatina*), die zusammen mit den beiden Rachenmandeln (*Tonsillae pharyngeales*) und der Zungenmandel (*Tonsilla lingualis*) den *Waldeyerschen Rachenring* bildet. Dieser besteht aus lymphoepithelialem Gewebe und dient als immunologische Barriere der oberen Atemwege.

Der Raum zwischen Lippen, Wangen und der oberen und unteren Zahnreihe bzw. Alveolarfortsätzen (*Procc. alveolares*) des Ober- und Unterkiefers (*Maxilla et Mandibula*) wird als Mundvorhof (*Vestibulum oris*) bezeichnet. Zwischen den eigentlichen Zahnreihen befindet sich die Mundhaupthöhle (*Cavum oris proprium*). Die gesamte Mundhöhle ist mit einem mehrschichtigen, unverhornten Plattenepithel ausgekleidet, durchzogen mit einer Vielzahl an kleinen oberflächlichen Speicheldrüsen (z. B. *Glandulae palatinae, buccales et labiales). *Tab. 1 gibt eine Übersicht der oralen mukösen und serösen Drüsengewebe.

**Table 1:** 

Anatomische Region	Drüse	Drüsentypus
*Mundboden*	Submandibularis	Gemischt, v. a. serös
	Sulingualis	Gemischt, v. a. mukös
*Zunge*		
Rand	Weber Blandin Nuhn	Gemischt (v. a. mukös)
Dorsal	Von Ebner	Gemischt (v. a. serös)
*Harter Gaumen*	–	Mukös
*Backen*	Parotis	Serös
	Bukkale Mukosa	Gemischt (v. a. mukös)
*Lippen*	–	Serös

Ausgefüllt wird die Mundhöhle weitgehend durch die Zunge (*Lingula*). Sie besteht im Wesentlichen aus quergestreifter Muskulatur. Auf ihrer Oberfläche findet sich neben kleinen Speicheldrüsen (*Glandulae linguales*) eine Vielzahl an gustatorischen und taktilen Sinneszellen. Im mittleren und hinteren Drittel ist sie am Zungenboden mit dem Mundboden und über die Zungenwurzel (*Radix linguae*) am Zungenbein (*Os hyoideum*) verwachsen.

**Figure 1:**
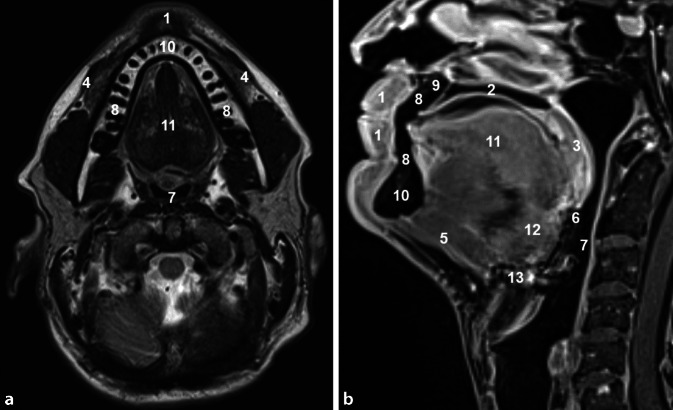

Einen Überblick über die Anatomie der Mundhöhle gibt Abb. 1.

## Pathologie

Die Veränderungen der Mundhöhle können aufgrund ihrer Histologie eingeteilt werden in oberflächliche und hyperplastische Läsionen, benigne und maligne Tumoren. Einen Überblick hierzu bietet Tab. 2.

**Table 2:** 

*Oberflächliche Läsionen*	
Embryonale Reste und Heterotypien	Fordy-Krankheit, Chievietz-Organ
Vesikulobullöse Erkrankungen	Herpes-simplex-Infektion, Herpes-zoster-Infektion, Coxsackie-Virus-Infektion, Herpangina, Pemphigus vulgaris, Pemphigus vegetans, paraneoplastischer Pemphigus, Schleimhaut-Pemphigoid, lineare IgA-Dermatose, Erythema exsudativum multiforme
Ulzeröse Läsionen	Aphthöse Stomatitis, Morbus Behcet, Reiter-Syndrom, Glossitis rhombica mediana, eosinophiles Ulkus, akute nekrotisierende ulzeröse Gingivitis, Wegener-Granulomatose, Tuberkulose
„Weiße Läsionen“	Candidose, Lichen planus, Lupus erythematodes, epitheliale Nävi, Raucherkeratose, Stomatitis nicotinica, *Haarzunge*, Haar-Leukoplakie, Lingua geographica, Friktionskeratose
Pigmentierungen	Amalgam-Tätowierung, lokalisierte melanotische Pigmentierung, Melanoakanthom, Pigmentnävi, prämaligne orale Melanosen, Morbus Addison, Peutz-Jeghers-Syndrom, Laugier-Hunziker-Syndrom, Raucher-Melanose, Medikamenten-assoziierte Pigmentierungen
*Hyperplastische Läsionen*	Fibröse Hyperplasien, papilläre Hyperplasien, generalisierte fibröse Gingiva-Hyperplasie, Morbus Crohn, orofaziale Granulomatose, chronische marginale Gingivitis, lokalisierte fibröse Gingiva-Hyperplasie, peripheres Riesenzellengranulom. Phylogenese Granulom, Zerealien-Granulom, Hyperplasie des Waldeyer-Rachenrings
*Benigne Tumoren*	Riesenzellenfibrom, verruciformes Xanthom, Hämangiome, Lymphangiom, benigne Nervenscheidentumoren (Neurofibrom, Schwannom, Neurofibromatose, multiple Neurome bei endokrinem Neoplasie-Syndrom), Granularzelltumor
*Maligne Tumoren*	Plattenepithelkarzinom, adenoidzystisches Karzinom, Adenokarzinom, Mukodermoidkarzinom, maligne Lymphome, andere seltene maligne Tumoren der Mundhöhle (z. B. orales Melanom, Ameloblastom, Metastasen etc.)

Oberflächliche und hyperplastische Läsionen können angeboren, entzündlicher bzw. autoimmuner oder infektiöser Genese sein. Am häufigsten sind vesikulobullöse, ulzeröse oder pigmentierte Prozesse des nichtverhornenden Plattenzellepithels der Mundschleimhaut. Entartungen sind möglich und häufig Ausgang für maligne Tumoren der Mundhöhle. Dennoch werden diese Veränderungen in aller Regel klinisch diagnostiziert und sind daher radiologisch üblicherweise nicht von größerem Interesse. In seltenen Fällen können einige Entitäten auch differenzialdiagnostisch für den Radiologen von Bedeutung sein. Bei jungen Patienten ist neben der Hyperplasie des lymphatischen Rachenrings differenzialdiagnostisch v. a. an Lymphome zu denken (s. unten).

Benigne Tumoren der Mundhöhle können sich von einer Vielzahl von Geweben ableiten. Im Vordergrund stehen Gefäße, Binde- oder Nervengewebe. Es handelt sich um proliferative Prozesse, die verdrängend wachsen können, jedoch meist keine Destruktion umliegenden Gewebes verursachen. Eine Entartung oder Metastasierung dieser Tumoren ist in aller Regel nicht zu erwarten. Klinisch relevant werden sie im Fall funktioneller Einschränkung, was auch eine radiologische Diagnostik im präoperativen Rahmen notwendig machen kann.

Die meisten malignen Tumoren der Mundhöhle sind epithelialen, glandulären oder lymphatischen Ursprungs. Das Plattenepithelkarzinom der Mundhöhle ist bei Weitem das häufigste Malignom der Mundhöhle. Es wird gefolgt von den malignen Tumoren der kleinen Speicheldrüsen und malignen Lymphomen. In Tab. 3 wird eine Übersicht über das TNM-Staging der malignen Tumoren der Mundhöhle gegeben.

**Table 3:** 

*Primärtumor (T)*		
T1	Tumor ≤2 cm (max. Durchmesser)	
T2	Tumor >2 cm und ≥4 cm (max. Durchmesser)	
T3	Tumor >2 cm und ≥4 cm (max. Durchmesser)	
T4a	Moderat fortgeschritten (Mandibula- oder Maxilla-Infiltration, Zungenmuskulatur, Kieferhöhle, Gesicht)	
4b	Sehr fortgeschritten (Kaumuskulatur, Schädelbasis, A. carotis interna!)	
*Lymphknotenbefall (N)*		
N1	Singulär, ipsilateral ≤3 cm (max. Durchmesser)	
N2a	Singulär, ipsilateral >3 cm ≤6 cm (max. Durchmesser)	
N2b	Multipel, ipsilateral ≤6 cm (max. Durchmesser)	
N2c	Bilateral oder kontralateral	<6 cm (max. Durchmesser)
N3		>6 cm (max. Durchmesser)
*Fernmetastasen (M)*		
M1	Fernmetastasen	

## Bildgebung

Aufgrund der anatomischen Zugänglichkeit steht bei der Diagnostik oberflächlicher Veränderungen der Mundhöhle die fachärztliche Inspektion und histopathologische Abklärung im Vordergrund. In Einzelfällen kann auch bei oberflächlichen Veränderungen der Mundschleimhaut eine weiterführende radiologische Diagnostik sinnvoll erscheinen.

Zur Abklärung tieferliegender oder raumfordernder Prozesse sollte zur Beurteilung der Ausdehnung bzw. Infiltration neben der klinischen und endoskopischen stets eine radiologische Untersuchung erfolgen. Hierzu stehen nach der noch gültigen, jedoch aktuell in Überarbeitung befindlichen S3-Leitlinie „Diagnostik und Therapie des Mundhöhlenkarzinoms“ von 2012 neben dem Ultraschall (US) die Computertomographie (CT) und Magnetresonanztomographie (MRT) zur Verfügung. Eine Überlegenheit von CT oder MRT in der Beurteilung von Primärtumoren der Mundhöhle oder zum Nachweis einer Knocheninvasion konnte nicht gezeigt werden. Falls ausgedehnter metallischer Zahnersatz vorliegt, was häufig der Fall ist, sollte der dünnschichtigen MRT gegenüber der CT der Vorzug gegeben werden, obgleich moderne Aufnahme- und Rekonstruktionsverfahren heute sowohl für die CT als auch die MRT eine deutliche Reduktion von Metallartefakten ermöglichen [3].

Eine Empfehlung zur CT- und MRT-Bildgebung des Oropharynx gibt Tab. 4 [4].

**Table 4:** 

CT	MRT
Injektion von Kontrastmittel i.v. (50 ml Omnipaque Iohexal 300) mit 2 ml/s, gefolgt von 50 ml NaCl mit 2 ml/s. Delay: 45–60 s. Zum Scan Patienten bei geschlossenen Lippen die Backen aufblasen lassen (zur besseren Differenzierung von Wangen/Lippen und Zunge). Scanbereich ab Sella turcica bis Aortenbogen. Im Fall störender Metallartefakte ggf. selektiver Re-Scan gewinkelt entlang der Mandibula	T1 axial und koronar mit 3,0 mm Schichtdicke/0,3 mm Schichtabstand, FOV („field of vision“) 180 mm. T2 axial und koronar fettgesättigt mit 3,0 mm Schichtdicke/0,3 mm Schichtabstand, FOV 180 mm. Axiale DWI (b500) mit 4,0 mm Schichtdicke/1,0 mm Schichtabstand, FOV 240 mm. T1 axial und koronar fettgesättigt nach Injektion von Kontrastmittel i.v. (Gadovist, adaptiert) mit 3,0 mm Schichtdicke/0,3 mm Schichtabstand, FOV 180 mm

Neuere Studien deuten darauf hin, dass auch der Ultraschall(US)-Diagnostik ein höherer Stellenwert eingeräumt werden sollte. Dies betrifft insbesondere Patienten, die einer MRT nicht zugänglich sind. Zugute kommen dem US die hohe Verfügbarkeit und Patientenverträglichkeit, die vergleichsweise geringen Kosten, die höhere Bildauflösung und das Fehlen von Metallartefakten, sodass er bei frühen Tumoren insbesondere der beweglichen Zunge und der bukkalen Weichteile eine ernstzunehmende Alternative darstellt. Limitiert wird der US durch allgemein beengte knöcherne Verhältnisse der Mundhöhle, aufgrund derer Tumoren in direkter Nachbarschaft der knöchernen Strukturen und im Bereich der hinteren Mundhöhle dem US schwer zugänglich sind [5].

Neben der lokalen Ausbreitungsdiagnostik sollte bei malignen Prozessen der Mundhöhle eine radiologische Umfelddiagnostik erfolgen. Dabei steht die Erfassung des Lymphknotenstatus im Vordergrund. Sie erfolgt in der Regel mittels CT- oder MRT-Bildgebung des Halses und der oberen Thoraxapertur. Der US bietet, auch ergänzend z. B. mittels kontrastgestütztem Ultraschall (CEUS) oder bildgesteuerter Feinnadelbiopsie (FNB), die Möglichkeit, die Spezifität der Untersuchung noch weiter zu steigern. Obgleich der US dabei wertvolle diagnostische Informationen wie feine Details (z. B. hiläre Strukturen, periphere Follikel oder Gefäßarchitektur) beisteuern kann, welche so mit der CT- oder MRT-Bildgebung in der Regel nicht erfasst werden können, wird er in der derzeit gültigen S3-Leitlinie aufgrund eingeschränkter Spezifität bei kleinen Lymphknoten, eingeschränkter Darstellung tiefliegender Lymphknoten und hoher Untersucherabhängigkeit zur alleinigen Beurteilung der Lymphknoten aktuell nicht empfohlen [3].

Bei lokal fortgeschrittenen Tumoren (Tab. 3) sollte zur Erfassung von Fernmetastasen eine CT des Thorax und ggf. abdominale US-Untersuchung angeschlossen werden. Bei lokal fortgeschrittenen Tumoren kann zum Ausschluss von Fernmetastasen vor funktionseinschränkenden Eingriffen zudem ergänzend eine Positronen-Emissions-Tomographie/CT (PET/CT) sinnvoll sein. Die PET/CT kann darüber hinaus auch zur Steigerung der diagnostischen Spezifität und Sensitivität des zervikalen Lymphknotenstagings beitragen. In der Primärdiagnostik des Lokalbefunds kommt der PET/CT hingegen kein gesonderter Stellenwert zu, wobei diese eine der CT und MRT überlegene Sensitivität bei der Erkennung von Rezidivtumoren aufweist [3].

Neben der Diagnosestellung und der Verlaufsbeurteilung wird die Bildgebung auch zunehmend zum intraoperativen Monitoring und für die intraoperative Navigation („augmented reality“) eingesetzt.

## Therapie

Die Therapie des Mundhöhlenmalignoms sollte im Rahmen einer interdisziplinären Zusammenarbeit erfolgen. Die primäre Behandlungsmethode von Mundhöhlenmalignomen basiert im Wesentlichen auf der Operation, Strahlen- und Chemotherapie. Je nach Ausmaß der Erkrankung erfolgt nach der Operation eine anschließende postoperative Strahlentherapie. Die wichtigsten Indikationen für eine postoperative Radiotherapie sind positive Resektionsränder und Kapselüberschreitung von Lymphknotenmetastasen, da diese als Prognosefaktoren deutlich schlechtere Ergebnisse vorhersagen. Eine begleitende Chemotherapie auf Cisplatin-Basis wird bei Plattenepithelkarzinomen zur postoperativen Strahlentherapie hinzugefügt. Weitere Indikationen für postoperative Strahlentherapie sind perineurale Invasion, Knocheninvasion, knappe (<5 mm) Resektionsränder, 2 oder mehr positive Lymphknoten oder ein fortgeschrittenes klinisches T‑Stadium (cT3–cT4). Einige Studien schließen auch eine Invasionstiefe von mehr als 5 mm als Indikation für postoperative Radiotherapie ein, obwohl dies bei Fehlen anderer Risikofaktoren umstritten ist [6].

Die Protonentherapie wird zunehmend zur Behandlung von Patienten mit bösartigen Tumoren eingesetzt. Aufgrund der physikalischen Eigenschaften der Protonen, die den Großteil ihrer Energie innerhalb des Bragg-Peaks deponieren, wird diese verwendet, um die Dosis an sensible Gewebestrukturen außerhalb des Tumorbereichs zu verringern. Insbesondere bei Tumoren der Nasennebenhöhlen kann die Wahrscheinlichkeit von Spätkomplikationen wie Schädigungen des zentralen Nervensystems und des Sehapparats verringert und gleichzeitig die lokale Kontrolle erhöht werden.

**Figure 2:**
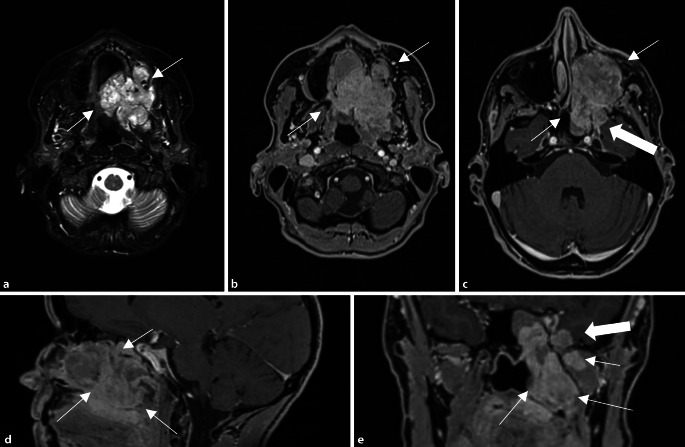

Bei Tumoren der Mundhöhle ist die Protonentherapie vor allem beim adenoidzystischen Karzinom indiziert, welches aufgrund seines perineuralen Ausbreitungsmusters häufig nicht komplett reseziert werden kann. Entlang der Hirnnerven V und VII können sich diese Tumoren bis nach intrakraniell ausdehnen (Abb. 2). In diesem Fall kann mit einer Protonentherapie eine hohe Strahlendosis in den Tumor gebracht und gleichzeitig das umliegende, strahlensensible Gewebe geschützt werden [7].

## Plattenepithelkarzinom

Das Plattenepithelkarzinom (PECA) ist für 2–4 % aller malignen Erkrankungen und für über 90 % der malignen Erkrankungen der Mundhöhle verantwortlich [8]. Es zeigt über die letzten Jahre eine steigende Inzidenz und wird mit etwa 1,4/100.000 (Frauen) bis 6,6/100.000 (Männer) angegeben, wobei die Inzidenz hohe regionale Unterschiede aufweist [9, 10].

Dies ist auch auf ein regional unterschiedliches Risikoverhalten der Bevölkerung in Bezug auf die beiden wichtigsten Risikofaktoren Tabakrauch und Alkoholkonsum zurückzuführen. Allein 75 % der PECA sind mit Tabakrauch vergesellschaftet [8, 9]. Weitere Risikofaktoren sind insbesondere virale Infektionen durch das humane Papillomavirus. Dabei steht der virale Subtyp HPV16 im Vordergrund [9]. In der überarbeiteten 4. Version der WHO-Klassifikation der Kopf-Hals-Tumoren von 2017 wird auf dieser Grundlage zwischen HPV-positiven und -negativen PECA unterschieden [11].

PECA entwickeln sich häufig auf dem Boden von Präkanzerosen wie der Erythroplakie oder Leukoplakie. Die meist ulzerösen Läsionen betreffen vorwiegend die Zunge, die Lippen und den Mundboden. In frühen Stadien ist das PECA meist schmerzlos und führt erst im fortgeschrittenen Stadium zu Symptomen wie Brennen oder Schmerzen. Daher bleibt es oft lange klinisch unbemerkt und wird meist erst im lokal fortgeschrittenen Stadium diagnostiziert [9].

In frühen Stadien (cT1 und cT2) besteht die Therapie in der kurativen Exzision und/oder einer Radiotherapie. In fortgeschrittenen Stadien (cT3 und cT4) wird eine Chemotherapie ergänzt [9]. Aufgrund zervikaler Lymphknotenmetastasen, wie sie in bis zu 80 % der Fälle auftreten, ist häufig eine radikale oder selektive Lymphadenektomie („cervical neck dissection“) nötig. Trotz der komplexen Therapieansätze liegt das 5‑Jahres-Überleben des PECA der Mundhöhle bei nur 40–50 %, in fortgeschrittenen Fällen unter 12 %, wobei HPV-negative prognostisch günstiger als HPV-positiven PECA sind [9, 11].

## Tumoren der kleinen Speicheldrüsen

Die zweithäufigsten Malignome der Mundhöhle sind Tumoren der kleinen Speicheldrüsen. Es handelt sich jedoch um insgesamt seltene Tumoren der Kopf-Hals -Region, die zusammengenommen für nur etwa 10–15 % aller Speicheldrüsentumoren verantwortlich sind. Im Gegensatz zu den meist gutartigen Tumoren der großen Speicheldrüsen sind die von den kleinen Speicheldrüsen ausgehenden Tumoren zu etwa 80 % bösartig [12]. Am häufigsten sind das Mukoepidermoidkarzinom, das polymorphe Adenokarzinom und das adenoidzystische Karzinom [13, 14].

### Mukoepidermoidkarzinom

Das Mukoepidermoidkarzinom (MEC) ist das häufigste Karzinom der kleinen Speicheldrüsen. Es ist für weniger als 10 % aller Speicheldrüsentumoren verantwortlich, jedoch für etwa 30 % aller malignen Tumoren der kleinen Speicheldrüsen [15, 16]. Frauen sind etwas häufiger betroffen als Männer (1,7:1) bei einem gehäuften Auftreten in der 3. bis 5. Lebensdekade [16].

Im Gegensatz zum PECA zeigt das MEC keine Assoziation zu Tabakrauch. Einige Studien deuten darauf hin, dass eine vorausgegangene Strahlenbelastung das Risiko erhöht, beispielsweise im Rahmen einer Strahlentherapie z. B. einer dermatologischen Erkrankung (z. B. Akne) oder einer Radiojodtherapie bei Erkrankungen der Schilddrüse [16].

Das Mukoepidermoidkarzinom (MEC) der kleinen Speicheldrüsen tritt am häufigsten im Bereich der Schleimhaut des harten und weichen Gaumens auf, wobei sich bis zu 18 % der intraoralen MEC retromolar finden. In absteigender Reihenfolge folgt die Mukosa von Wangen, Zunge, Lippen und Mundboden. Eine begleitende knöcherne Arrosion ist häufig, es wurden jedoch auch primär intraossäre MEC beschrieben. Klinisch präsentiert sich das MEC meist als schmerzlose Schwellung, kann sich jedoch auch schmerzhaft, infiltrierend, ulzerös oder fluktuierend äußern [14].

Anatomisch zugängliche MEC ohne Metastasierung werden kurativ reseziert. Häufig wird aufgrund knöcherner Mitbeteiligung und lokaler Infiltration eine erweiterte Resektion, beispielsweise unter Teilresektion der Mandibula oder Mundbodenmuskulatur notwendig. Eine selektive „neck dissection“ sollte soweit möglich nur bei hochgradig entdifferenzierten MEC erfolgen. Bei mittel- oder niedriggradigen MEC wird sie aufgrund der guten Prognose nicht empfohlen. Eine adjuvante Radiotherapie kann die Prognose bezüglich etwaiger Lymphknotenmetastasen verbessern [14].

### Polymorphes Adenokarzinom

Der ehemals als *polymorphes niedriggradiges Adenokarzinom* (PNGA) beschriebene Tumor der kleinen Speicheldrüsen wird nach der neuen 4. WHO-Klassifikation der Kopf-Hals-Tumoren von 2017 mit dem *kribriformen Adenokarzinom der kleinen Speicheldrüsen* (KAC) zusammengefasst unter dem Begriff *polymorphes Adenokarzinom* (PAC). Das PNGA ist mit 41 % der zweithäufigste maligne Tumor der kleinen Speicheldrüsen; für das KAC wurden hingegen nur wenige Fälle in der Literatur beschrieben [11]. Die Inzidenz des PNGA wird mit etwa 0,05/100.000 angegeben, wobei Frauen gegenüber Männern in etwa doppelt so häufig betroffen sind, vorrangig ab einem Alter von 40 bis 79 Jahren [13].

Das PNGA betrifft meist den harten und weichen Gaumen, das KAC hingegen meist Zungengrund, seltener Wangenschleimhaut retromolar, Gaumen, Oberlippe oder Tonsillen. Nur ein Bruchteil der Tumoren äußert sich primär durch klinische Symptome wie Schmerz, Ulzerationen, Blutungen oder schlechtsitzende Prothesen. Meist fällt der Tumor durch eine einfache lokale Schwellung auf. Vermutlich aufgrund der besseren Sichtbarkeit wird dabei meist das PNGA etwas früher diagnostiziert als das KAC [13].

Ein weiterer Hauptunterschied zwischen dem PNGA und dem KAC besteht im jeweiligen Metastasierungsmuster. Nur 1 von 10 Patienten mit PNGA zeigt Lymphknotenmetastasen, aber 7 von 10 Patienten mit KAC. Dies mag auch mit der meist späteren Diagnosestellung einhergehen. Demgegenüber zeigen jedoch 4 % der Patienten mit PNGA parenchymatöse, dann meist pulmonale, Fernmetastasen, wohingegen solche beim KAC nur in einem einzigen Fall beschrieben wurden [13].

Sowohl für das PNGA als auch für das KAC steht therapeutisch die ausgedehnte Lokalresektion im Vordergrund. Dennoch wird, ähnlich dem adenoidzystischen Karzinom aufgrund früher perineuraler Tumorausdehnung nur in etwa einem Drittel der Fälle eine R0-Resektion erreicht. Daher wird häufig eine adjuvante Radiotherapie nötig. Die Chemotherapie ist hingegen meist nur in palliativen Fällen bei primär inoperablem Tumoren indiziert. Im postoperativen Verlauf sollten die Patienten über mindestens 15 bis 20 Jahre kontrolliert werden, um Lokalrezidive früh zu erkennen, da diese häufig aggressiver verlaufen als der Primärtumor [13].

### Adenoidzystisches Karzinom

Beim adenoidzystischen Karzinom (ACC) kann zwischen dem kribriformen, dem tubulären und dem soliden Typ unterschieden werden, wobei Letzterer mit einer schlechteren Prognose assoziiert ist. ACC wurden früher auch aufgrund ihrer Histologie als Zylindrome beschrieben und sind mit etwa 1 % aller Kopf-Hals-Tumoren und etwa 10 % aller Speicheldrüsenmalignome relativ selten. Betroffen sind Frauen wie Männer v. a. in der 4. bis 6. Lebensdekade [17].

**Figure 3:**
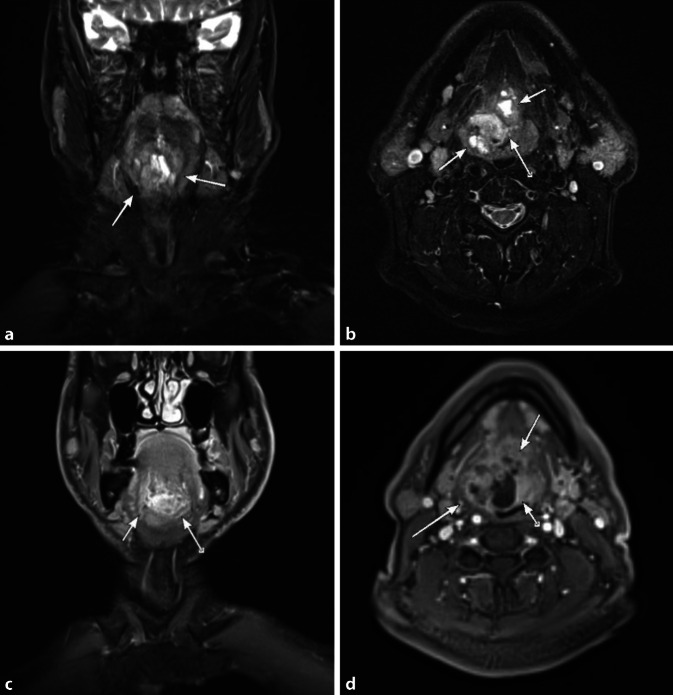

Ein Beispiel für ein histologisch gesichertes ACC zeigt Abb. 3.

Etwa ein Drittel der ACC finden sich in den kleinen und großen Speicheldrüsen, hauptsächlich in der Mundhöhle und sinunasal. Sie können jedoch auch von mukösem Drüsengewebe des oberen Respirationstrakts, von den Tränendrüsen, der Brust- oder Prostatadrüse und von der Lunge ausgehen. Lymphknotenmetastasen sind untypisch, wohingegen die hämatogene Metastasierung, insbesondere in die Lunge, charakteristisch ist [17].

Das ACC zeigt im Vergleich zu den anderen Speicheldrüsentumoren eine frühzeitige hämatogene Metastasierung. Klinisch stehen Schmerzen und fokalneurologische Symptome infolge ihrer perineuralen Ausbreitung im Vordergrund. Dabei wird aber auch ein subklinisches Wachstum mit frühzeitiger Metastasierung beobachtet. Die Tumorgröße hat hier keinen Einfluss auf die Wahrscheinlichkeit einer schon bestehenden hämatogenen Metastasierung [17]. Bei einer Tumorgröße >4 cm ist allerdings die Wahrscheinlichkeit der perineuralen Tumorausdehnung größer als bei kleineren Tumoren. Die Prognose wird dann wie im Fall einer verzögerten Diagnosestellung bzw. protrahiertem Therapiebeginn entsprechend schlechter. Rezidive könne auch bis zu 20 Jahre nach Diagnosestellung auftreten [17, 18].

Die Prognose hängt im Wesentlichen von der R0-Resektionsmöglichkeit ab. Die 10-Jahres-Überlebensrate variiert und beträgt, je nach Literatur zwischen 20 % bzw. 50–70 % [17, 18]. Auch bei der Bildgebung steht das perineurale Tumorwachstum im Vordergrund. Dieses kann jedoch prinzipiell auch bei anderen Kopf-Hals-Tumoren beobachtet werden. Bei der Therapie stehen die chirurgische Resektion und Bestrahlung einschließlich Protonentherapie im Vordergrund, wobei die alleinige Strahlentherapie als unzureichend gilt [17].

## Maligne Lymphome

Maligne Lymphome sind nach den epithelialen Karzinomen und den malignen Tumoren der kleinen Speicheldrüsen die dritthäufigste Malignität der Mundhöhle. Es handelt sich um eine heterogene Gruppe von Malignomen, die in die beiden Hauptgruppen der Hodgkin (HL) und Non-Hodgkin Lymphome (NHL) unterteilt wird [10]. Zusammen sind NHL und HL für etwa 3 % aller malignen Erkrankungen verantwortlich, wobei sich nur etwa 3–4 % im Bereich der Mundhöhle manifestieren. Das NHL ist dabei mit einer Inzidenz von etwa 1,7/100.000 (Frauen) bis 2,2/100.000 (Männer) häufiger als das HL mit 0,3/100.000 (Frauen) bis 0,4/100.000 (Männer) [1, 10].

Im Bereich der Mundhöhle steht das diffus großzellige B‑Zell-NHL (DLBCL) mit etwa 40 % im Vordergrund, gefolgt vom kleinzelligen NHL und dem Burkitt-Lymphom. Zu den Risikofaktoren zählen neben genetischen Faktoren jeweils in erster Linie virale Infektionen. Das EBV zeigt eine hohe Assoziation mit malignen Lymphomen und kann in 60–80 % der DLBCL und 50 % der Burkitt-Lymphome nachgewiesen werden. HIV-Patienten haben ein gegenüber der Normalbevölkerung etwa 100-fach erhöhtes Risiko, an einem Lymphom zu erkranken, wobei das DLBCL 70–80 %, und das Burkitt-Lymphom 7–20 % aller HIV-assoziierten Fälle betrifft [10].

Klinisch stellen sich maligne Lymphome der Mundhöhle oft unspezifisch dar. Häufig äußern sie sich durch Schmerz, Schwellungen oder Missempfindungen. Morphologische Veränderungen finden sich vorwiegend im Bereich des Waldeyer-Rachenrings, gefolgt von Veränderungen der Speicheldrüsen und der Maxilla. Die knöchernen Veränderungen können dabei radiologisch unscheinbar erscheinen und allein in einer erhöhten Strahlentransparenz des Knochens bestehen [10].

Therapie und Prognose der malignen Lymphome sind stark vom jeweiligen histopathologischen Subtyp abhängig. Erschwerend kommt hinzu, dass aufgrund der unspezifischen Symptomatik häufig zunächst auf eine vermutete bakterielle Infektion hin behandelt wird. Eine verzögerte Diagnosestellung kann dabei die Prognose zusätzlich verschlechtern. Insgesamt zeigen die meisten malignen Lymphome jedoch ein gutes Ansprechen auf eine auf den jeweiligen Subtyp abgestimmte Chemotherapie, ggf. in Kombination mit einer Radiotherapie. Eine chirurgische Exzision ist in aller Regel nicht notwendig [10].

## Fazit für die Praxis

- Das Spektrum der Mundhöhlentumoren ist groß. Dabei sind anlagebedingte Störungen von oberflächlichen Veränderungen (vesikulobullöse Erkrankungen, ulzeröse Läsionen, *weiße Läsionen*, Pigmentierungen) und tumorösen Veränderungen zu unterscheiden.
- Bei Raumforderungen können hyperplastische Läsionen von echten Neoplasmen unterschieden werden.
- Die Unterscheidung zwischen malignen und benignen Tumoren kann häufig schon mittels klinischer Untersuchung und histopathologisch nach Biopsie vorgenommen werden.
- Bei differenzialdiagnostischen Überlegungen, der exakten Tumorausdehnung einschließlich Tumorgrenzen und dem exakten Staging sowie für Verlaufsbeurteilungen unter und nach Therapie spielt die Radiologie und insbesondere die moderne Bildgebung eine wichtige Rolle.
